# Monthly Reports

**Published:** 1803-06-01

**Authors:** J. Ricards

**Affiliations:** 68, Hatton Street


### MONTHLY REPORTS.

*Cases admitted under the Care of the Surgeon of the Finsbury Dispensary, St. John's Square, Clerkenwell, from April* 10, *to May* 10, 1803.

| | | | |
|---|---|---|---|
| Phlegmone | 2 | Gonorrhœa | 2 |
| Erysipelas | 1 | Scrophula | 5 |
| Abscessus Artuum | 9 | Hernia | 1 |
| Mastodynia | 2 | Steatoma | 1 |
| Herniæ Humoralis | 2 | Hæmorrhois | 2 |
| Ulcera Artuum | 12 | Hydarthus Ischii | 1 |
| Lippitudo | 2 | Incontinentia Urinæ | 1 |
| Fistula in Ano | 1 | Strictura | 1 |
| Fractura Claviculæ | 3 | Eruptiones | 5 |
| Vulnera | 3 | Vacciola | 63 |
| Contusiones | 8 | | |
| Lues | 3 | Total | 128 |

In the last Reports some cursory observations were made on the good effects of electricity on disease; I shall, however, hope to be excused for again introducing that subject, by the relation of some well-marked cases. in which that power has proved itself a preservative of the limbs and lives of the patients.

The cases which I now allude to were diseased knee joints.

D. R. a person of about 30 years of age, had had at different times the venereal disease in its constitutional form, for which he had taken much mercury, without any regularity, nor conducted by the advice of any professional person: some months since he applied to me, on account of a disease of the knee joint, which had commenced during the action of mercury, and at the time when he had venereal pains in the bones, and which he therefore attributed to the same cause. The knee was very much enlarged, perfectly rigid, and extremely painful; I was not of opinion that it was altogether a symptom of the venereal disease, but rather a rheumatic affection, to which disease he had been previously very much subject, and which predisposition had been very much augmented by exposure to all the variations of atmosphere during the action of mercury; but still however, other venereal symptoms existed, as enlargement of the tibia, and pain there, and ulcerated throat. I therefore administered mercury, so as to produce a gentle and moderate action, by small quantities quantities of hydrarg. muriat. internally, and used frictions of the ointment to the knee. In the course of a few weeks all the truly-marked venereal symptoms disappeared, but the knee became worse. Blisters were used, fomentations, poultices, and every variety of topical application, as likewise various internal means, but without success; indeed, with an aggravation of the disease; the knee being now enlarged to an astonishing size, and his health gradually decreasing from the constant excess of pain.— In this state I began to use electricity, by small shocks, passed in every direction through the joint, and repeated every day. From the first week, the progress of the disease stopped, and it soon began to decrease, till, at the end of two months, no vestige remained, except some slight thickening of the ligaments, which, however, produced little impediment to motion.

The other case was in a person, not a patient of mine, but whom I saw once during the worst period of the disease, and whose symptoms and their progress I can relate, from the testimony of two very respectable surgeons of this metropolis. The patient was a young woman of about 25, and the disease was a true scrophulous white swelling; it had existed for a long time. She had conformed to the advice of many eminent surgeons, without benefit; she became hectic, and amputation was advised as the only preservative of her life. She in this state applied to one of the above surgeons, in order to undergo the operation; but her health was so reduced, that he thought she could hardly support it, as she laboured under a profuse diarrhœa. During the administration of some medicines which were given to abate that symptom, he passed some gentle shocks through the knee; which being daily repeated, seemed to relieve her pain considerably; all applications were omitted, except a linseed poultice; her health amended, the knee gradually lessened, and in a few months became perfectly well, except some slight stiffness of the joint.

In the last Report I announced my intention of submitting some observations founded on experiment, of the action of Galvanism on the healthy and diseased state of the animal body, and compared with the effects of electricity.

The accurate knowledge of the action of that principle, will, I doubt not, add much to our powers in the healing art; but such knowledge cannot be gained but by a very careful investigation and comparison of facts, and therefore some considerable time must elapse, before such observations can be in a state fit to be submitted to the public eye.

68, *Hatton Street.* J. RICARDS.

